# Aromatase Is a Direct Target of FOXL2: C134W in Granulosa Cell Tumors via a Single Highly Conserved Binding Site in the Ovarian Specific Promoter

**DOI:** 10.1371/journal.pone.0014389

**Published:** 2010-12-20

**Authors:** Nicholas I. Fleming, Kevin C. Knower, Kyren A. Lazarus, Peter J. Fuller, Evan R. Simpson, Colin D. Clyne

**Affiliations:** 1 Prince Henry's Institute of Medical Research, Clayton, Victoria, Australia; 2 Department of Biochemistry and Molecular Biology, Monash University, Victoria, Australia; Cincinnati Children's Research Foundation, United States of America

## Abstract

**Background:**

Granulosa cell tumors (GCT) of the ovary often express aromatase and synthesize estrogen, which in turn may influence their progression. Recently a specific point mutation (C134W) in the FOXL2 protein was identified in >94% of adult-type GCT and it is likely to contribute to their development. A number of genes are known to be regulated by FOXL2, including aromatase/*CYP19A1*, but it is unclear which are direct targets and whether the C134W mutation alters their regulation. Recently, it has been reported that FOXL2 forms a complex with steroidogenic factor 1 (SF-1) which is a known regulator of aromatase in granulosa cells.

**Methodology/Principal Findings:**

In this work, the human GCT-derived cell lines KGN and COV434 were heterozygous and wildtype for the FOXL2:C134W mutation, respectively. KGN had abundant FOXL2 mRNA expression but it was not expressed in COV434. Expression of exogenous FOXL2:C134W in COV434 cells induced higher expression of a luciferase reporter for the ovarian specific aromatase promoter, promoter II (PII) (−516bp) than expression of wildtype FOXL2, but did not alter induction of a similar reporter for the steroidogenic acute regulatory protein (StAR) promoter (−1300bp). Co-immunoprecipitation confirmed that FOXL2 bound SF-1 and that it also bound its homologue, liver receptor homologue 1 (LRH-1), however, the C134W mutation did not alter these interactions or induce a selective binding of the proteins. A highly conserved putative binding site for FOXL2 was identified in PII. FOXL2 was demonstrated to bind the site by electrophoretic mobility shift assays (EMSA) and site-directed mutagenesis of this element blocked its differential induction by wildtype FOXL2 and FOXL2:C134W.

**Conclusions/Significance:**

These findings suggest that aromatase is a direct target of FOXL2:C134W in adult-type GCT via a single distinctive and highly conserved binding site in PII and therefore provide insight into the pathogenic mechanism of this mutation.

## Introduction

The forkhead transcription factor FOXL2 has recently emerged as a critical regulator of ovarian function. It is one of the first molecular markers of ovarian development [1] and its ablation inhibits ovarian differentiation of the embryonic bipotential gonad [2], [3], [4], [5]. It is one of a handful of genes that is mutated in premature ovarian failure (POF) and recently, a specific somatic mutation in FOXL2 (C134W) was discovered in more than 94% of ovarian adult-type GCT [6], [7]. The mutation is likely to facilitate adult-type GCT development and may possibly be the tumor's primary cause.

Despite the biological importance of FOXL2, many details of its action remain unknown or are the subject of conflicting reports. A number of putative target genes for FOXL2 have been identified including genes involved in steroidogenesis (e.g. *STAR*, *CYP17* and aromatase), inflammation (e.g. *NFAT* and *PTGS2/COX2*) and apoptosis or detoxification (e.g. *MNSOD*) [8], [9]. Interestingly, aromatase was found to be up-regulated by FOXL2 in COS7 cells and ovine granulosa cells [10] but down-regulated by FOXL2 in CHO cells [11]. FOXL2 has been reported as a negative regulator of the *STAR* gene [12], therefore it is likely that the consequence of its binding to gene promoters is context or co-factor dependent. The influence of the FOXL2:C134W mutation on the regulation of these genes is unknown.

The DNA binding site of FOXL2 is also controversial. Some reports claim that it binds sites similar to the consensus forkhead element [13], [14], while another study suggests that it binds a sequence similar to an extended nuclear receptor half-site (TCAAGGTCA) also known as the SF-1 response element (SFRE) [15]. These findings are intriguing because forkhead factors are known to serve as co-regulators of nuclear receptors and recently, FOXL2 was reported to bind and co-regulate SF-1 via its forkhead domain [14], [16].

The molecular consequence of the FOXL2:C134W mutation is also unclear. C134 is located within wing 2 of the forkhead domain, which is a divergent component of the domain's secondary structure and of uncertain function. Although the forkhead domain as a whole has DNA binding function, it is unclear whether wing 2 contributes to DNA binding [17], [18], [19], [20] or facilitates other molecular interactions [7], [21]. Importantly, a recently published characterization of the FOXL2:C134W mutation has suggested that it does not markedly alter the regulation of a number of known FOXL2 target regions [22].

In this work, we have characterized the two GCT cell lines, KGN and COV434, with respect to FOXL2 mutation status and expression of aromatase and SF-1. We report that the mutation alters FOXL2 regulation of the aromatase promoter but not that of the StAR promoter. We provide evidence that the effect of the mutation on aromatase regulation is not altered by the presence of SF-1 and show that it does not alter interactions between FOXL2 and SF-1 or its close homologue, LRH-1. We identify a highly conserved forkhead element in the aromatase promoter which is bound by FOXL2 and finally, we demonstrate that this site alone confers its sensitivity to FOXL2:C134W.

## Results

### KGN cells have robust expression of aromatase mRNA and the FOXL2:C134W mutation; COV434 cells have little aromatase mRNA expression, are wildtype for FOXL2, but do not express it

Two well-characterized GCT cell lines, KGN and COV434, have been the subject of a number of comparative studies due to their contrasting characteristics [23], [24], [25], [26]. The KGN cells have a spindle-like morphology and form a monolayer at confluence [27] whereas the COV434 cells are spherical, grow in clusters and their growth is inhibited by cell-cell contact [28], [29]. The KGN cells are known to harbor FOXL2:C134W [30]. We sought to establish the FOXL2 genotype of the COV434 cell line and characterize the mRNA expression of aromatase and its known regulator SF-1 in both cell lines.

**Figure 1:**
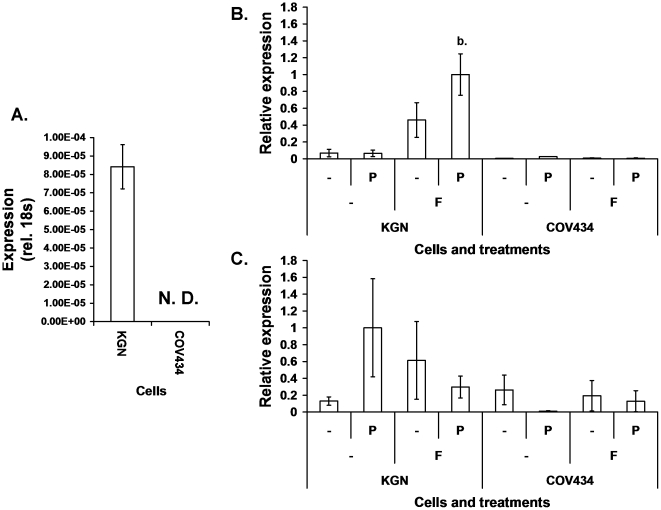
The KGN and COV434 cell lines differ in expression of FOXL2 and aromatase mRNA. (A) RT-qPCR measures of FOXL2 mRNA in KGN (heterozygous for FOXL2:C134W) and COV434 cells (wildtype) relative to expression of 18s rRNA, N. D.: not detected. Mean of three experiments, error bars: ±SEM. (B) RT-qPCR measures of mRNA expression for aromatase following 24hr treatment with separate and combined FSK and PMA, F: FSK, P: PMA. Means of three experiments, error bars: ±SEM, differences in expression were tested by 1-way ANOVA followed by Dunnett's post hoc analysis, relative to the untreated control for each cell line. Indications of significance are a. = p<0.05, b. = p<0.01 and c. = p<0.001. (C) RT-qPCR measures of mRNA expression for SF-1 within the cDNA set generated for panel B. Differences in expression between the two cell lines and the combined treatments were tested by 2-way ANOVA followed by Tukey's post hoc analysis considering all possible comparisons. No comparison was significantly different (difference between cell lines p<0.097).

Direct sequencing of genomic DNA confirmed that the KGN cells were heterozygous for FOXL2:C134W but revealed that the COV434 cells had wildtype sequence (data not shown). However, reverse transcribed-quantitative PCR (RT-qPCR) analysis of the FOXL2 transcript showed that where the KGN had robust FOXL2 mRNA expression, the COV434 cells had little or no FOXL2 expression (Fig. 1A).

In normal granulosa cells, aromatase is known to be up-regulated by follicle stimulating hormone (FSH) signaling via cAMP and in cooperation with the protein kinase C (PKC) pathway. Aromatase and SF-1 expression were measured by RT-qPCR in KGN and COV434 cells, following separate and combined treatment with forskolin (FSK) and phorbol myristate acetate (PMA) (Fig. 1B and 1C). Strikingly, KGN cells had robust expression of aromatase mRNA upon FSK treatment which was increased by combined treatment with PMA, whereas in the COV434 cell line, aromatase mRNA expression was either very low or absent (Fig. 1B). SF-1 mRNA expression had greater variance and the difference in expression between the lines was not significant (Fig. 1C).

### C134W augments FOXL2 induction of aromatase but not StAR

**Figure 2:**
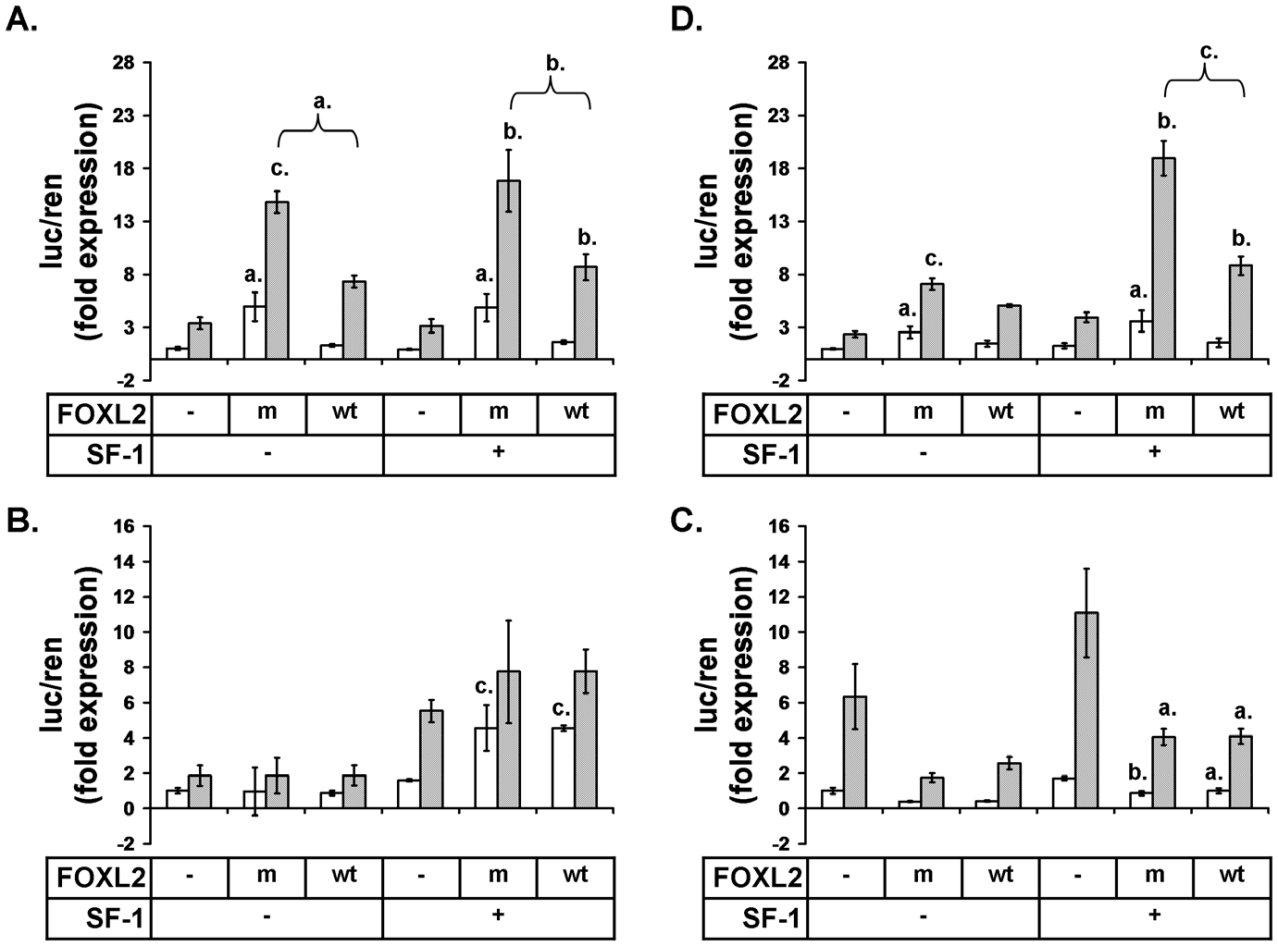
The C134W mutation increases FOXL2 stimulation of aromatase but does not alter FOXL2 regulation of StAR. Luciferase assays were performed in COV434 and COS7 cells with PII −516bp-luciferase and StAR −1300bp-luciferase 48hr after co-transfections with FOXL2:wt (wt), FOXL2:C134W (m), SF-1 and pcDNA3.1+ (−) as indicated, and either treated (grey bars) or untreated (white bars) with combined FSK and PMA. (A) Aromatase reporter in COV434 cells. (B) StAR reporter in COV434. (C) StAR reporter in COS7 cells. (D) Aromatase reporter in COS7 cells. Means of four (COV434) or three (COS7) experiments, error bars: ±SEM, differences in expression were tested by 1-way ANOVA followed by Tukey's post-hoc analysis considering all possible comparisons, indications of significance are a. = p<0.05, b. = p<0.01 and c. = p<0.001, and they relate to comparisons of FOXL2 transfections with the closest matched pcDNA3.1+ control or when indicated with braces, to differences between matched FOXL2:wt and FOXL2:C134W transfections. Differences between SF-1 transfection alone and matched pcDNA3.1+ controls were not significant for all panels.

The finding that COV434 cells do not express FOXL2 provided the opportunity to examine the influence of ectopic FOXL2 expression on the regulation of a firefly luciferase reporter for the ovarian specific promoter of aromatase, PII (PII −516bp-luciferase) [31]. We expressed FOXL2 and FOXL2:C134W together with PII −516bp-luciferase in COV434 cells. The FOXL2 and FOXL2:C134W expression constructs were equally expressed in COV434 cells (Fig. S1) and both up-regulated aromatase reporter expression with FOXL2:C134W stimulating twice as much expression as the wildtype protein (Fig. 2A). In parallel, we tested the influence of the mutation on the regulation of a firefly luciferase reporter for the StAR promoter (StAR −1300bp-luciferase) [32], as StAR was previously reported to be down-regulated by FOXL2 [12]. Interestingly, FOXL2 expression up-regulated StAR reporter expression contrary to the previous report, but there was no difference in the stimulation by FOXL2 and FOXL2:C134W (Fig. 2B). These data therefore suggest that the FOXL2 mutation alters aromatase stimulation but not that of StAR. Given the unexpected result for StAR, we repeated the experiment in COS7 cells and found that in that setting, FOXL2 down-regulated StAR reporter expression as previously reported (Fig. 2C). Aromatase was differentially regulated by FOXL2 and FOXL2:C134W in COS7 cells, as seen in the COV434 cells, but was more sensitive to SF-1 expression (Fig. 2D). In summary, these results suggest that the C134W mutation increases stimulation of aromatase by FOXL2 in GCT, but does not alter its regulation of StAR.

### FOXL2 binds SF-1 and inhibits its transcriptional activation, and it also binds LRH-1, but these interactions are not altered by C134W

**Figure 3:**
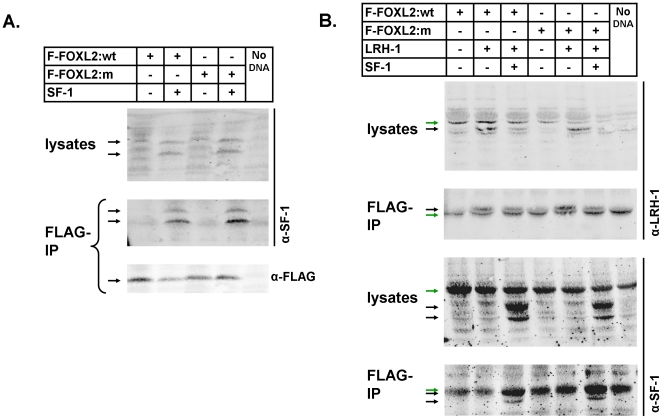
FOXL2 binds SF-1 and also LRH-1, but these interactions are not altered by the C134W mutation. Co-immunoprecipitation was performed for SF-1 and LRH-1 via FLAG-tagged FOXL2:wt and FOXL2:C134W (m) 24hr following co-transfection into COV434 cells, as indicated. (A) SF-1 alone. The use of the same antibody across more than one blot is indicated by a vertical bar. Two arrows are used to indicate SF-1 because this antibody typically detects two bands for SF-1 which is thought to result from post-translational modification of the protein. (B) SF-1 and LRH-1 alone and combined. Black arrows indicate proteins detected by antibody, green arrows indicate non-specific bands.

Given that the SF-1 and LRH-1 proteins are key regulators of aromatase [33], [34] and it has been reported that SF-1 forms a physical complex with FOXL2 [14], [16], we tested whether human FOXL2 could also form a complex with human LRH-1, whether the mutation prevented interactions between FOXL2 and SF-1 or LRH-1, or whether it conferred preferential binding to one of them. Human SF-1 was clearly pulled down by immunoprecipitation of both wildtype FOXL2 as reported and also mutant FOXL2 (Fig. 3A). LRH-1 was pulled down in a similar manner to SF-1 by FOXL2 immunoprecipitation and again this was not affected by the C134W mutation (Fig. 3B). Finally, co-expression of both SF-1 and LRH-1 with FOXL2 and FOXL2:C134W did not result in an altered binding or preferential binding of the co-factors to the FOXL2 proteins (Fig. 3B). These findings together confirm that FOXL2 binds SF-1 and show that it also binds its homologue LRH-1, but suggest that the manner in which it does so is not affected by the presence of the C134W mutation.

### A highly conserved putative forkhead element resides in the ovarian aromatase promoter

**Figure 4:**
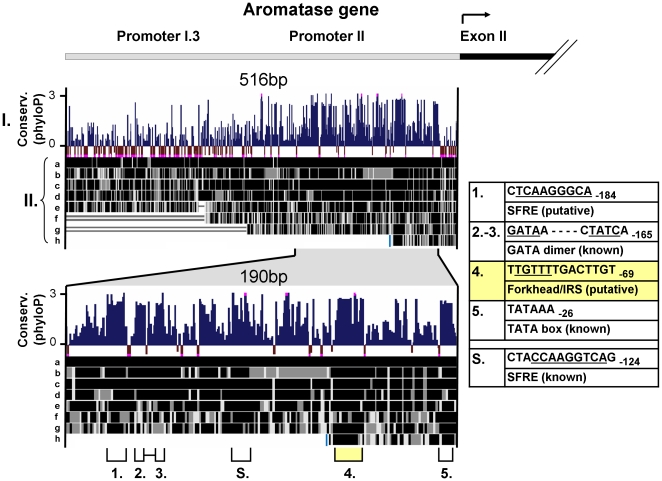
A highly conserved putative forkhead element resides in the ovarian aromatase promoter (PII). Sequence conservation analysis was performed on the human aromatase promoter (−516bp) and a highly conserved putative FOXL2 binding site was identified. (I.) PhyloP conservation analysis of 32 placental mammals. Scores exceeding 3 or −0.5 are truncated and indicated in pink. Conserved sequences containing at least 4 contiguous base pairs with phyloP scores >2 were selected (numbered 1–5). The conserved sequences and putative or known regulatory elements within them are displayed to the right. A well described but less conserved functional SFRE is also indicated (S.) [34]. (II.) Multiz alignment based on 46 vertebrates demonstrating high conservation of the putative FOXL2 binding site. Species displayed: a. monkey (Rhesus), b. mouse, c. dolphin, d. dog, e. possum, f. chicken, g. lizard, h. frog. Light blue bar indicates limit of available sequence for frog.

The nature and context of DNA binding sites of transcription factors give insight into the action of those transcription factors. We sought to determine where in PII, FOXL2 bound to regulate the promoter. FOXL2 is a highly conserved protein [35] with a fundamental role in ovarian development and this suggests that significant target sites may also be conserved. We performed in parallel, inter-species conservation analysis of the promoter and mapping of binding sites for known transcription factors (Fig. 4). Five highly conserved sequences were identified, of which one contained a putative forkhead element (−82 to −69). The other conserved regions contained the TATA box (−31 to −26), a previously described binding site for dimeric GATA transcription factors (−179 to −165) [36], [37] and an uncharacterized putative SFRE (−193 to −184). Two further putative forkhead elements were present in the promoter, but these did not reside within conserved regions. We selected the highly conserved putative forkhead element for further study.

### FOXL2 binds to the putative forkhead element in the aromatase promoter and disruption of the element blocks increased stimulation by FOXL2:C134W

**Figure 5:**
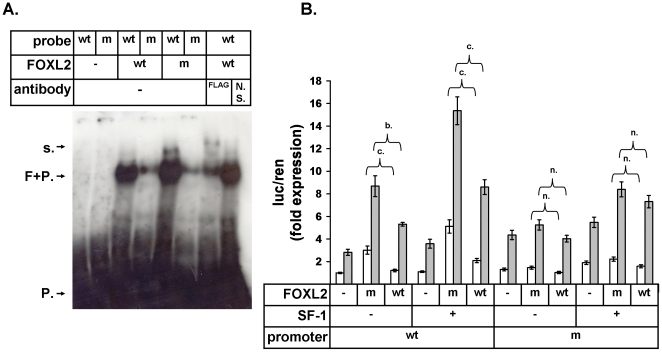
FOXL2 binds the conserved element and mutation of the site blocks increased stimulation by FOXL2:C134W. (A) EMSA analysis of *in vitro* translated FOXL2:wt (wt) and FOXL2:C134W (m) binding of putative conserved binding site in aromatase PII. 38bp γ^32^P-ATP end-labelled probes centered on the putative binding site were used with 3 consecutive thymidine bases changed to 3 guanine bases in the mutant probe (m) (refer to Fig. 6), N. S.: non-specific antibody, FP: FOXL2 bound to probe, P: unbound probe, s: supershifted band. The experiment was performed three times graphically analyzed (Fig. S2). (B) Luciferase assays using reporters for the wildtype aromatase promoter (−516) and for the promoter mutated at the putative FOXL2 binding site (m) in the same manner as the EMSA probe. Assays were performed 48hr following transfection of COV434 cells as indicated and either treated (grey bars) or untreated (white bars) with combined FSK and PMA. Means of four experiments, error bars are ±SEM, differences in expression were tested by 1-way ANOVA followed by Tukey's post-hoc analysis considering all possible comparisons, indications of significance are a. = p<0.05, b. = p<0.01, c. = p<0.001 and n. = not significant.

**Figure 6:**
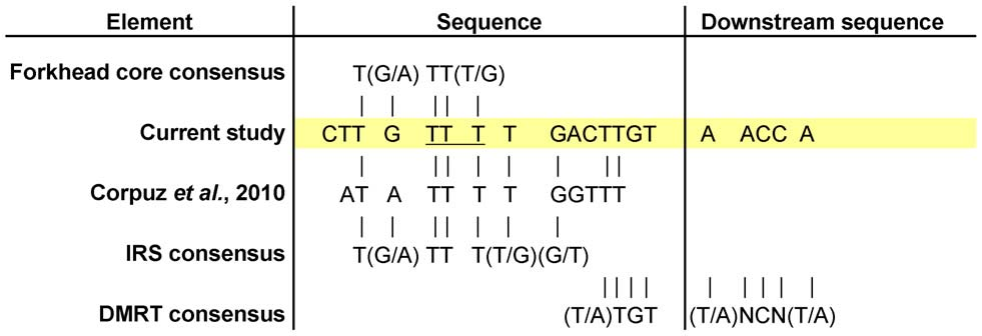
Comparison of the identified putative FOXL2 binding site with various relevant sequences. The identified conserved sequence (highlighted in yellow) was aligned with the FOXL2 binding site in the *FSHB* promoter described by Corpuz *et al.* [44] and the consensus sequences for **the forkhead** [**45**] **, IRS** [**46**] **and DMRT** [**53**] **elements.** **Bases mutated in EMSA and luciferase reporter assays (** **Fig. 5** **) are underlined.**

To determine whether the identified putative forkhead binding element in PII was bound by FOXL2 we performed an EMSA using 38bp probes centered on the site (Fig. 5A). We generated a mutant probe in which the three consecutive thymidine bases characteristic of the forkhead element core sequence (refer to Fig. 6) were changed to three guanines. Both the wildtype FOXL2 and FOXL2:C134W proteins bound the wildtype DNA probe robustly and bound the mutated probe at a reduced level (Fig. 5A and Fig. S2). The addition of FLAG antibody to a binding reaction of wildtype FOXL2 with wildtype probe generated a supershifted band confirming that the detected complexes included the FOXL2 protein (Fig. 5A, lane 7). These data suggest therefore that the identified element is bound by FOXL2 and that both the wildtype and mutant proteins bind it in a similar manner. Interestingly, a minor band positioned above the major complex band appeared to have more intensity in the FOXL2:C134W lane (Fig. 5A, lane 5) and this may indicate that the mutation was promoting the formation of a higher order complex for a small portion of the protein.

To test whether the identified forkhead element was a functional regulator of the promoter and whether it could explain the increased stimulation of PII −516bp-luciferase by the FOXL2:C134W (Fig. 2) we generated a mutant version of the reporter, in which the putative element was mutated in the same manner as in the EMSA probes above. When FOXL2 or FOXL2:C134W was expressed with the reporters, differential expression was detected with the wildtype reporter but not with the mutated reporter (Fig. 5B). These data therefore indicate that this element is required for the increased stimulation of the wildtype reporter by FOXL2:C134W. Importantly, the mutant and wildtype aromatase reporters were equally stimulated by FSK/PMA treatment (Fig. 5B) indicating that the mutant promoter was still functional and regulated in a normal fashion by other regulatory elements within the promoter (e.g. cAMP response elements).

## Discussion

The potentially oncogenic FOXL2:C134W mutation is remarkable for its molecular specificity and therefore it provides an opportunity to define a new pathogenic mechanism. Here we confirmed that the KGN cell line is heterozygous for the FOXL2:C134W mutation and found that it has robust FOXL2 expression. The COV434 cell line was wildtype for FOXL2 but had low or no expression. FSK/PMA treatment induced aromatase robustly in KGN cells but not in COV434 cells. These findings combined with a dramatic difference in cellular morphology [27], [29] show that the KGN and COV434 differ greatly in their biology and they of course suggest that COV434 were not derived from an adult-type GCT but more likely from a rarer juvenile-type GCT. In support of this, the KGN cells had been generated from a 73 year old patient [27] and the COV434 cells were taken from a 27 year old [28], [29]. Significantly, the finding that COV434 cells lack FOXL2 together with reports by Kalfa and co-workers, that juvenile-type GCT have low or aberrant FOXL2 expression [38], [39] suggest that an alteration of FOXL2 function is a feature of both the adult and juvenile GCT subtypes.

The FOXL2:C134W mutation was found to increase FOXL2 induction of an aromatase reporter but not one for StAR, indicating that the mechanism of FOXL2 action on the two promoters is different and that aromatase may belong to an undefined subset of genes that are influenced by the mutant protein. Notably, the mutation appeared to circumvent the requirement for FOXL2 stimulation of aromatase on concurrent PKA and PKC signaling (Fig. 2A). The StAR promoter was previously reported to be negatively regulated by FOXL2 and we confirmed that this occurred in the non-GCT cell line, COS7. These findings mean that FOXL2 action is both promoter and cell type specific and implies that FOXL2 interacts with multiple co-regulatory factors that result in either stimulation or repression of its target genes.

Few co-regulators have been described for FOXL2. It has been reported to form complexes with SMAD3 [21], DPC103/GEMIN3 [40], ERα [41] and SF-1 [14], [16], and to be subject to sumoylation by Ubc9 [42], [43]. One or all of these interactions could be relevant to the regulation of aromatase in various settings, but in the ovary the relationship between FOXL2 and SF-1 and its close homologue LRH-1, are of clear importance and have relevance to concurrent PKA and PKC signaling [33], [34]. A number of results presented here however, argue against the interaction of FOXL2 with SF-1 or its homologue LRH-1 being involved in the pathogenicity of FOXL2:C134W. First, the co-immunoprecipitation of SF-1 or LRH-1 with FOXL2 was not altered by the presence of the mutation (Fig. 3). It is possible that a more subtle affect may be detected with a more sensitive method (e.g. Biacore) but it is unclear that such would explain the pathological impact of the substitution. Second, our luciferase assays demonstrated that SF-1 increased reporter expression cooperatively and to the same extent when combined with either wildtype or mutant FOXL2 (Fig. 2 and 5) and this happened with both the aromatase and StAR reporters (Fig. 2). Interestingly, in COV434 cells, FOXL2 did not stimulate the StAR promoter without SF-1 being co-transfected suggesting that its stimulatory action here was entirely dependent on SF-1 (Fig. 2B). Thirdly, when the putative forkhead site in PII, which conferred its sensitivity to FOXL2:C134W, was disrupted in the reporter, SF-1 still stimulated it to the same extent as the wildtype reporter and did so in cooperation with either the mutant or wildtype FOXL2 (Fig. 5B). We conclude therefore, that it is unlikely that the pathogenicity of FOXL2:C134W relates to an alteration of its binding of SF-1 or, with less certainty, LRH-1.

The sequence and context of DNA elements bound by transcription factors give insight into the action of those factors. Here we identified a highly conserved region in PII containing a forkhead element that conferred its sensitivity to increased stimulation by FOXL2:C134W (Fig. 4 and 5). Our approach was validated by the co-identification of both the gene's TATA box and a previously characterized dimeric GATA binding site stretching from −179 to −165 [37]. The high conservation of the element, extending to lizards and frogs (Fig. 4), suggests that it may be of fundamental importance in the regulation of aromatase by FOXL2. Given that FOXL2 is one of the earliest markers of ovarian differentiation, its action via this element may be crucial to female gonadal development. As we hoped, a number of features of the identified element illuminate the role of FOXL2 in the regulation of aromatase.

The first observation we have made is that the conserved region extends well beyond the forkhead element it contains. Strikingly, the region is of a similar length to a putative FOXL2 binding site recently identified by Corpuz and co-workers in the promoter of the FSHβ subunit gene (*FSHB*) (Fig. 6) [44]. In their study, sequential mutational analysis revealed that FOXL2 binding was reduced by mutation of any of the 12 consecutive base pairs at their site. Our conserved region is 14bp long and has high sequence similarity to their sequence (Fig. 6) and importantly, both sites include a run of four thymidines instead of the normal three found in the consensus forkhead binding sequence [45]. Our study therefore, appears to corroborate Copuz' finding that FOXL2 binds this sequence motif and implies that the pathogenicity of FOXL2:C134W may relate to sequences like these.

A second observation we have made of our identified element is that it is also a putative insulin response sequence (IRS), which as a group have the consensus T(G/A)TTT(T/G)(G/T) [46] (Fig. 6). This is highly significant as IRS are well documented to be bound by members of the FOXO subfamily of forkhead proteins [47], [48], [49] and members of this family play a key role in the regulation of ovarian follicle development [50]. For example, the Foxo3 knockout mouse has a POF phenotype not dissimilar to the Foxl2 knockout [51], [52]. Hence, it possible that the action of FOXL2 on the aromatase promoter is integrated with that of the FOXO proteins via this element.

A third observation is that the identified forkhead element is immediately flanked by a potential binding site for members of the doublesex and mab-3 related transcription factor (DMRT) family so that the core sequence of this binding site (A/T,TGT) [53] is contained within the highly conserved 14bp region (Fig. 6). This is of significance, because the DMRT proteins are homologues of the *Drosophila* gene that controls sex determination in that species [54]. In mammals, DMRT1 is a key positive regulator of testis development [55] and other members of the DMRT family also exhibit male specific expression [56], [57]. It is possible therefore that the identified sequence is not only key to female gonadal development but is regulated in both female and male sex determination.

**Figure 7:**
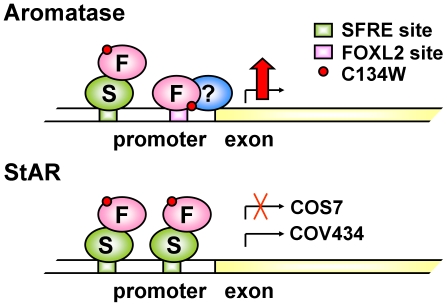
Proposed model for FOXL2:C134W action on the aromatase and StAR promoters in GCT. FOXL2 stimulates both aromatase and StAR, but C134W only increases stimulation of aromatase. Given the sequence of the putative binding site in PII, it is likely that FOXL2 directly binds the promoter of aromatase at that site, recruiting an unknown protein(s), and this is altered by the mutation, whereas on the StAR promoter, FOXL2 acts indirectly, perhaps via SF-1/LRH-1. However, other explanations are possible (see discussion). F: FOXL2, S: SF-1/LRH-1.

Finally, a fourth and key observation made of the identified sequence is that it is actually a forkhead sequence and not a binding site for members of a different protein family. FOXL2 forms complexes with other transcription factors (e.g. SF-1) and it is likely to bind DNA both directly and indirectly (this is probably why Benayoun and co-workers found that FOXL2 bound an approximate SFRE [15]). This means that the FOXL2:C134W mutation, which resides within the DNA binding domain of FOXL2, is likely to confer its pathogenic effect while that domain is directly bound to DNA, and therefore it is either directly altering DNA binding or it is altering an associated protein-protein interaction. Critically, we saw no evidence that the mutation directly alters DNA binding in our EMSA blots (Fig. 5A and Fig. S2). With this in mind a model can be proposed for the action of FOXL2:C134W on the aromatase and StAR promoters (Fig. 7). On the aromatase promoter, FOXL2 binds directly to DNA at the identified forkhead site and the mutation alters a relevant protein-protein interaction (relevant to concurrent PKA and PKC signaling but not involving SF-1 or LRH-1) and in turn, alters transcription; however on the StAR promoter, FOXL2 binds DNA indirectly via SF-1, and here the mutation can not alter transcription (Fig. 7). Intriguingly, a candidate for the unknown protein may be another molecule of FOXL2 itself. If FOXL2 were to form a dimer, this interaction may be altered by the mutation and affect target transcription. Dimerization of FOXL2 is supported by recent *in silico* modeling based on crystal structures of other forkhead protein family members [22] and by our observation of a minor band with increased intensity in the FOXL2:C134W lanes of our EMSA blots (Fig. 5A). However, the observed band represents only a small portion of the protein and other scenarios remain equally possible, for example, the mutation may alter a protein-protein interaction with another member of the forkhead transcription factor family, perhaps a FOXO protein, which binds to the DNA element instead of FOXL2. In support of this idea, the element is a putative IRS as mentioned, and an analogous regulation of a FOXO protein by further forkhead family member, FOXG1, was reported to operate on the p21/Cip1 promoter in glioblastoma cells [58]. Further work is therefore required before the exact molecular details of the action of FOXL2 at the identified site are established.

Nevertheless, these studies reveal that aromatase is a target of FOXL2:C134W and identify the genomic element through which it operates. The element is of a type known to be bound by FOXO proteins, and through its conservation, it appears to be of fundamental importance in the ovary. The translation of these findings to other genes will illuminate the functional consequence of FOXL2:C134W in GCT.

## Materials and Methods

### Cell lines

KGN cells [27] were maintained in DMEM/HamsF12 media (Invitrogen, Carlsbad, CA; Gibco) supplemented with 10% fetal bovine serum (FBS), 2mM L-glutamine and penicillin/streptomycin antibiotics (Invitrogen; Gibco) in a 37°C incubator with 5% CO_2_. COV434 cells [28], [29] were maintained in the same manner except that the base media was DMEM (Invitrogen; Gibco). FSK (Sigma-Aldrich, St Louis, MO) and PMA (Sigma-Aldrich) were used at final concentrations of 25µM and 4nM, respectively.

### Molecular biology and expression constructs

The *FOXL2* gene was amplified and then sequenced with a 3130×l Genetic Analyzer (Life Technologies, Carlsbad, CA; Applied Biosystems) using primers previously described [7]. The FOXL2 expression construct was generated by sub-cloning from the IMAGE consortium clone 6572303 into pcDNA3.1+ with or without sequence encoding a FLAG tag. Site-directed mutagenesis of the FOXL2 expression constructs and the aromatase luciferase reporter was performed with the QuickChange II kit (Agilent Technologies, Santa Clara, CA; Stratagene) and appropriate oligonucleotides. The expression construct for SF-1 was a kind gift from Dr. Ken-ichirou Morohashi and the construct for LRH-1 was previously reported [33].

### RT-qPCR

RNA was prepared using the Trizol reagent (Agilent Technologies; Stratagene) according to the manufacture's instructions and cDNA was generated using AMV reverse transcriptase (Promega, San Luis Obispo, CA) with poly-dT primers. RT-qPCR was performed on a Rota-Gene RG-3000 (Qiagen, Germantown, MD; Corbett Life Science) using previously described primer sets for SF-1 [59], aromatase [60] and FOXL2 [7].

### Luciferase assays

Cells were plated at 15% confluence and transiently co-transfected with constructs of interest and either PII −516bp-luciferase [31], a modified version of that construct as described below, or StAR −1300bp-luciferase [32], using the Fugene 6 reagent (Roche, Basel, Switzerland). Cells were washed and/or treated with combined FSK and PMA at 24hrs and luciferase assays were performed at 48hrs using the Dual-Luciferase Reporter Assay System (Promega) and measured on a Victor 2 plate reader (Perkin Elmer, Waltham, MA; Wallac). Firefly luciferase signals were compared to *Renilla* luciferase signals generated by co-transfection of a *Renilla* luciferase expression construct [61].

### Co-immunoprecipitation and Western analysis

For Western analysis of expression from the FLAG-tagged FOXL2 expression constructs, lysates were generated by the addition of lysis buffer containing 1% Igepal CA-630 (Sigma-Aldrich), 24hr following transfection. The proteins were separated on a SDS-PAGE gel and visualized by standard methods using anti-FLAG antibody (Sigma-Aldrich; M2). For co-immunoprecipitation, lysates were generated in the same manner and then mixed with Anti-FLAG M2 Affinity Gel (Sigma-Aldrich). The beads were washed three times with 20 volumes of lysis buffer and then boiled in denaturing sample loading buffer. The remaining proteins were separated on a SDS-PAGE gel and visualized using commercially available antibodies for LRH-1 (Abcam, Cambridge, MA; ab18293) and FLAG (Sigma-Aldrich; M2), and with anti-sera for SF-1 that was kindly provided by Dr. Ken-ichirou Morohashi. Densitometry was performed with EZQuantGel (EZQuant, Tel-aviv, Israel).

### Homology analysis

DNA homology analysis was performed via the University of California Santa Cruz (UCSC) Genome Browser [62] employing the BLAST-like alignment tool (BLAT) [63] to align the human aromatase promoter sequence with phyloP genome conservation data based on 32 placental mammalian genomes [64] and with Multiz genome alignments based on 46 vertebrate genomes [65]. The phyloP score is displayed in units of −log p-value of a null hypothesis of neutral evolution for the given base pair. Transcription factor recognition site predictions were generated with MatInspector [66].

### EMSA

Probes of the sequences 5′-tttcttgggcttccttgttttgacttgtaaccataaat-3′ and 5′- tttcttgggcttccttggggtgacttgtaaccataaat-3′ (mutant) were prepared by end labeling annealed oligos with γ^32^P-ATP using T4 Polynucleotide Kinase (Promega) and purification with MicroSpin™ G-50 Sephadex columns (Amersham Biosciences). EMSA were performed by incubating the probes with *in vitro* translation products generated with the TNT Quick transcription/translation kit (Promega) either with or without appropriate antibodies. The complexes were separated on non-denaturing polyacrylamide gels and visualized by drying and subsequent overnight exposure to autoradiography Hyperfilm MP at −80C (GE Healthcare). Densitometry was performed with EZQuantGel (EZQuant, Tel-aviv, Israel).

## Supporting Information

Figure S1 FOXL2:wt and FOXL2:C134W constructs were expressed equally following transfection. The FOXL2:wt (wt) and FOXL2:C134W (m) constructs were transiently transfected into COV434 cells and lysates were prepared 24hr later. The lysates of three experiments were visualized together on a single Western blot using anti-FLAG antibody. (A.) Western blot. (B.) Densitometry of bands detected in B. Mean of three experiments, error bars are ±SEM.
(0.85 MB TIF)

Figure S2 FOXL2:wt and FOXL2:C134W bound to the promoter probe and bound less to the mutated probe in a similar manner. Densitometry of three EMSA blots including the example shown in Fig. 5A. Mean of three experiments, error bars are ±SEM, m. mutant, wt. wildtype, N. S. non-specific.
(0.50 MB TIF)
