# Supplementary figures and images for: Aromatase Is a Direct Target of FOXL2: C134W in Granulosa Cell Tumors via a Single Highly Conserved Binding Site in the Ovarian Specific Promoter
Source: PLoS One. 2010 Dec 20;5(12):e14389. doi: 10.1371/journal.pone.0014389 (PMC3004790; doi:10.1371/journal.pone.0014389)

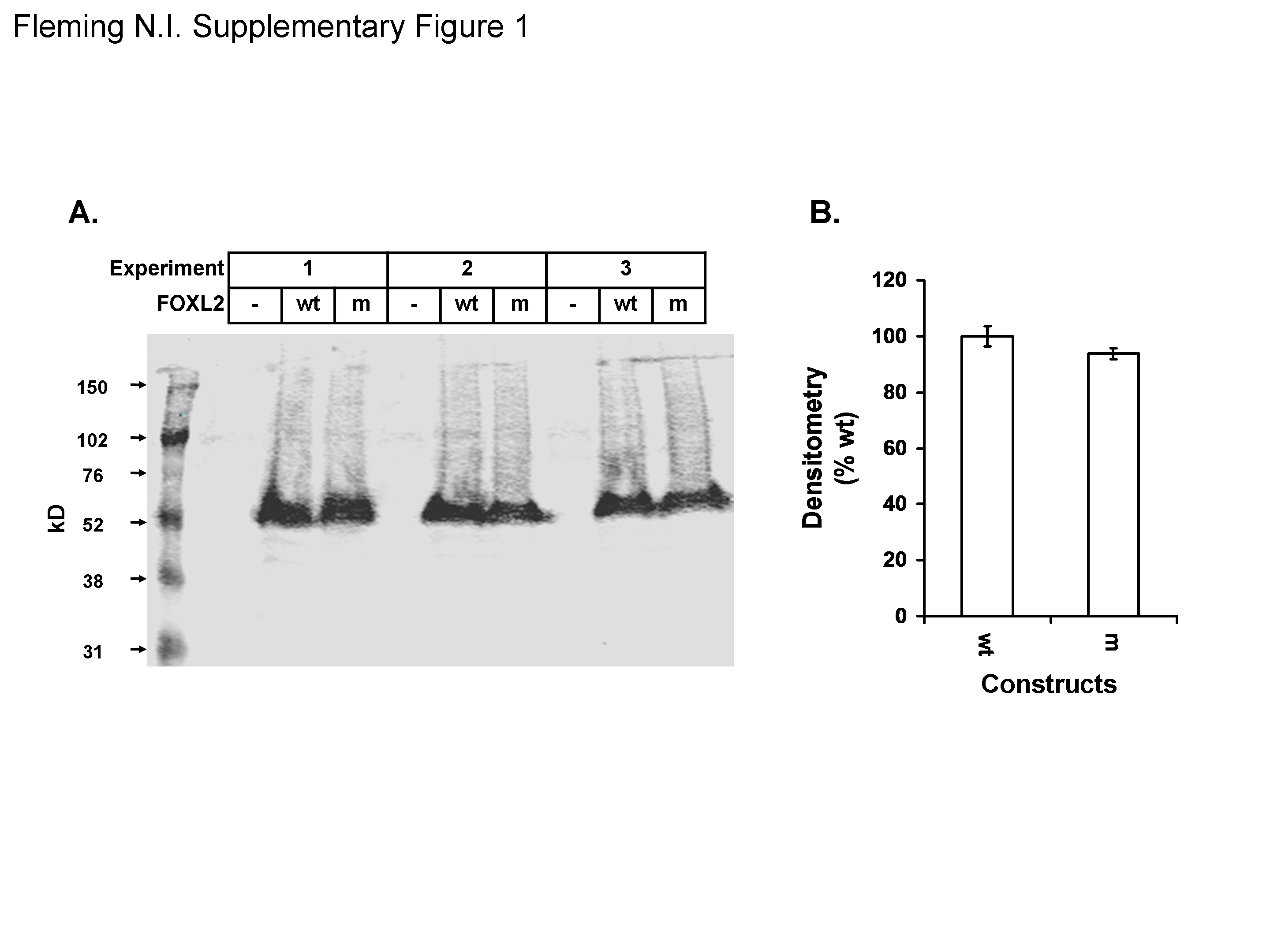

Supplement: Figure S1 — FOXL2:wt and FOXL2:C134W constructs were expressed equally following transfection. The FOXL2:wt (wt) and FOXL2:C134W (m) constructs were transiently transfected into COV434 cells and lysates were prepared 24hr later. The lysates of three experiments were visualized together on a single Western blot using anti-FLAG antibody. (A.) Western blot. (B.) Densitometry of bands detected in B. Mean of three experiments, error bars are ±SEM. (0.85 MB TIF) [file pone.0014389.s001.tif]

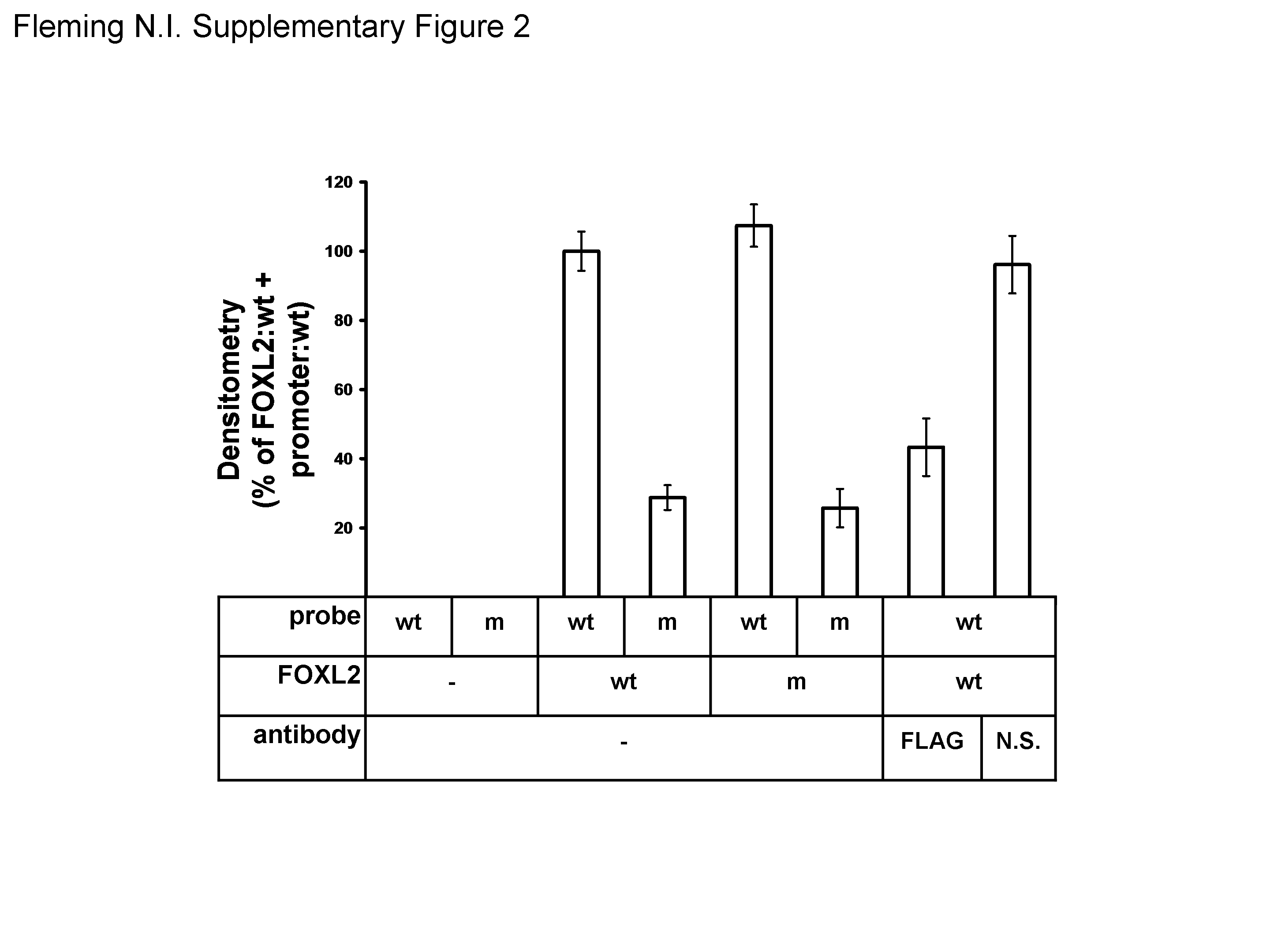

Supplement: Figure S2 — FOXL2:wt and FOXL2:C134W bound to the promoter probe and bound less to the mutated probe in a similar manner. Densitometry of three EMSA blots including the example shown in Fig. 5A. Mean of three experiments, error bars are ±SEM, m. mutant, wt. wildtype, N. S. non-specific. (0.50 MB TIF) [file pone.0014389.s002.tif]
